# A Novel Mach-Zehnder Interferometer Using Eccentric-Core Fiber Design for Optical Coherence Tomography

**DOI:** 10.3390/s18051540

**Published:** 2018-05-13

**Authors:** Qiao Xiong, Xinglin Tong, Chengwei Deng, Cui Zhang, Pengfei Wang, Zhiyuan Zheng, Fang Liu

**Affiliations:** 1 National Engineering Laboratory for Fiber Optic Sensing Technology, Wuhan University of Technology, Wuhan 430074, China; 18202768827@163.com (Q.X.); dchw@163.com (C.D.); zc@whut.edu.cn (C.Z.); 13545031181@163.com (P.W.); manjyun3180@gmail.com (Z.Z.); liumingfang303@outlook.com (F.L.); 2 School of Information Engineering, Wuhan University of Technology, Wuhan 430074, China

**Keywords:** eccentric core fiber, optical coherence tomography, Mach-Zehnder interferometer

## Abstract

A novel Mach-Zehnder interferometer using eccentric-core fiber (ECF) design for optical coherence tomography (OCT) is proposed and demonstrated. Instead of the commercial single-mode fiber (SMF), the ECF is used as one interference arm of the implementation. Because of the offset location of the eccentric core, it is sensitive to directional bending and the optical path difference (OPD) of two interference arms can be adjusted with high precision. The birefringence of ECF is calculated and experimentally measured, which demonstrates the polarization sensitivity of the ECF proposed in the paper is similar to that of SMF. Such a structure can replace the reference optical delay line to form an all-fiber passive device. A mirror is used as a sample for analyzing the ECF bending responses of the system. Besides, four pieces of overlapping glass slides as sample are experimentally measured as well.

## 1. Introduction

Optical coherence tomography (OCT) is a tomographic imaging technique with micron-scale resolution, which has revolutionized biomedicine since its introduction to imaging biological tissues such as human skin, retina and blood vessels [1,2,3,4]. OCT system is based on a classic optical measurement technique known as low-coherence interferometry [5]. A low-coherence source is used in the OCT system and the most common interferometer for OCT is a Mach-Zehnder interferometer (MZI). The broadband light is split into the reference and the sample arms of the MZI. The recombined light in the detection fiber only coherently interfered when the path lengths in the sample and reference arms are matched to within the short coherence length [6,7,8,9]. All OCT systems require setting the length of the reference arm [5]. Time domain OCT requires adjusting the length of the reference arm for real-time imaging. Fourier domain OCT requires setting the length of the reference arm for phase match with the sample arm and depth scanning [10]. Kushal et al. reported a swept-source OCT system for pediatric bronchoscopy and they adjusted the reference arm delay length for providing a relative delay [11]. The reference setting method predicts a much greater roll off than they actually observe when samples are placed at great depths. Thus, designs accurate to reference arm optical length variations are desirable and efforts have been made to solve the problem. For instance, a collimator and adjustable delay retro-reflector in the reference arm was reported [12]. The optical path length of the reference arm was adjusted by the preset reference mirror position as precisely as less than 0.1 micrometers. The location of the mirror was driven by a motor. It has useful properties in adjusting the optical path in experimental measurement, but with the downside that the motor can’t work without a voltage supply. A topology including a fiber stretching auto-correlator with Faraday mirrors was also reported and has been successfully applied in an OCT endoscopic system [13,14]. A DC-voltage controlled piezofiber delay element provided a simple solution with ~4 mm adjustment range using ~350 V. Such a method can adjust the OPD with a large regulation range, but to some extent the complex operation and high DC-voltage supply limit the engineering applications. Consequently, the creation of a simple, easy to manufacture and cost-effective OCT system design having sufficient technical performance remains a challenge.

In this paper, a MZI using eccentric-core fiber (ECF) design for OCT is proposed. Instead of the commercial single-mode fiber (SMF), the ECF is used as one interference arm of the implementation. The birefringence of ECF is calculated and experimentally measured, which demonstrates the polarization sensitivity of the ECF proposed in the paper is similar to that of SMF. The polarization effects in the setup could be ignored. Because of the elongation and compression of the ECF during directional bending process [15,16], the optical path in ECF changes continuously. Therefore, the OPD of two arms can be adjusted with high precision just using a mechanical bending equipment. Such a structure is passive and replace the reference delay line to form an all-fiber configuration with simple structure. Besides, four pieces of glass slides as sample are experimentally used for tomography as well, which demonstrates implementation of the system.

## 2. Materials and Methods

### 2.1. Theory

**Figure 1:**
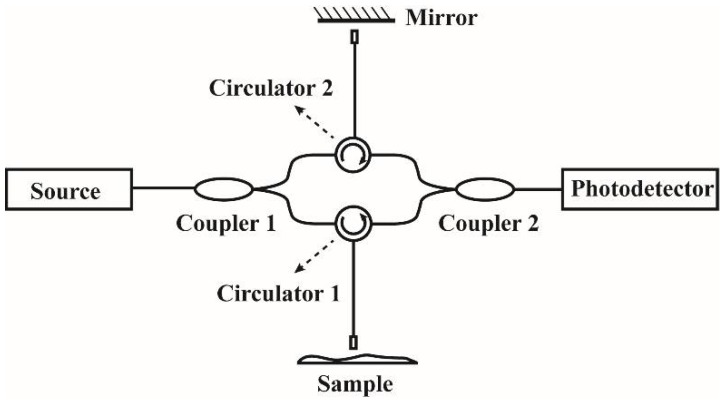
The schematic diagram of an OCT system using all-fiber MZI.

The theoretical analysis of the OCT system is based on an all-fiber MZI, as depicted in Figure 1. The incident light source is divided by a 1 × 2 coupler into a reference beam $E_{R}$ and a signal beam $E_{S}$ which travel different distances in the two interferometer arms. 

Circulators are used in both sample and reference arms to redirect the back reflected light to a 2 × 2 fiber coupler, whose splitting ratio is 50:50. The electric field of the interferometer output is the sum of the signal and reference fields $E_{R}\;+\;E_{S}$, and a photodetector is used to measure the output intensity I(k), which is proportional to the square of the total resulting interference field:

(1) $$I\left(k\right)\sim {\left|E_{r}\right|}^{2}+{\left|E_{s}\right|}^{2}+2\cdot E_{r}\cdot E_{s}cos\left[2\cdot k\cdot \left(l_{r}-l_{s}\right)\right]$$

On the right hand of this formula, ${\left|E_{r}\right|}^{2}$ and ${\left|E_{s}\right|}^{2}$ are the DC components which would not exist for balanced detection; $2\cdot E_{r}\cdot E_{s}cos\left[2\cdot k\cdot \left(l_{r}-l_{s}\right)\right]$ is the cross-correlation term, which contributes to the interference signal. The OPD between $E_{R}$ and $E_{S}$ is $l_{r}-l_{s}$. The recombined light in the output of the interferometer only coherently interferes when the path lengths in the sample and reference arms were matched to within the short coherence length interference. More specifically, $l_{r}-l_{s}$ should be less than the coherence length of the source $l_{c}$. Fringe contrast drops as the OPD between the reference and sample arms increases and gets close to the source coherence length. To select the imaging depth of interest for the enface imaging, the interferometer with an adjustable arm length is required.

**Figure 2:**
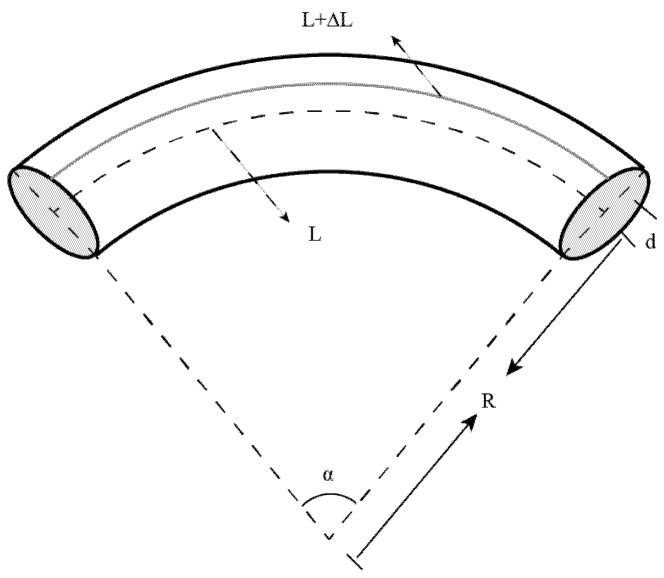
The schematic view of a curved ECF.

The core of ECF deviates from the central axis, which makes the light beam travel off axis rather than along the center axis. Because of the elongation and compression of the ECF during the bending process, the optical path in ECF changes continuously. Figure 2 depicts the schematic view of a curved ECF. When the ECF is bending, the equation can be written as:

(2) L/R=(L+ΔL)/(R+d)

where L is the length of the ECF before ECF bending, ΔL is the length variation of the ECF, R is the radius of curvature and d is the distance between the eccentric core and the neutral plane. ΔL can be expressed as:

(3) ΔL=(L·d)/R=L·d·C

where C is the curvature of the bent ECF.

### 2.2. Experimental Setup

**Figure 3:**
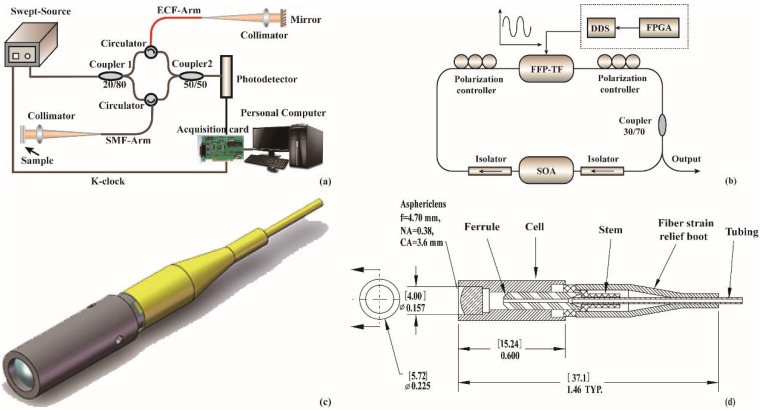
(**a**) The OCT setup using the ECF; (**b**) The schematic diagram of swept-source laser; (**c**) The CAD diagram of the collimator and (**d**) The SolidWorks diagram of the collimator.

The schematic diagram of the proposed OCT system is shown in Figure 3a, which is based on a fiber-optic MZI. The OCT system consists of a 20.65 mW wavelength custom-made swept source centered at 1310 nm with a sweep rate of 2 kHz, and a sweep range of 60 nm. The schematic diagram of swept source is depicted on Figure 3b. The swept source laser utilizes a SOA (IPSAD1304, InPhenix, Inc., Livermore, CA, USA) as gain medium. The broadband optical signal generated by the SOA is transmitted in a single direction by the isolator to ensure the direction of transmission. Polarization controller is used to adjust the light signal polarization and then are transmitted into the FFP-TF (FFP-TF, Micron Optics, Inc., Atlanta, GA, USA) filter for filtering processing. The driver signal of the filter is generated by the homemade function signal generator. The function signal generator is mainly composed of a FPGA (XC6SL8) controlled Digital Synthesizer (DDS: AD9834, Norwood, MA, USA), which produces a sinusoidal signal of 2 kHz to drive FFP-TF. A MZI whose one interference arm using ECF compensates the OPD between the reference light and sample light. The SMF-arm is the sample arm which used pigtailed fiber collimators with a light illuminated to a sample through the lensed SMF probe. The collimation package consists of a stainless-steel housing and an aspheric lens. The CAD and SolidWorks diagrams of the collimator are shown in the Figure 3c,d. The ECF-arm is the reference arm with fiber end cut flatly to form reference reflector because of the low reflectivity (~4%) of the air-silica reflective surface. A variable attenuator is inserted between the ECF-arm and the circulator for matching the optical power of two interferometer arms to improve the system signal-to-noise ratio (SNR). The light from the swept source is split 20:80 by coupler 1. Two beams of light are transmitted into the SMF-arm (80%) and ECF-arm (20%) through circulators, respectively. The same circulators are used to redirect the reflected/scattered light of the SMF-arm and ECF-arm into the 50:50 output coupler 2, where the interferometric mixing is achieved and then the interferometric signal is sent to a 30 GHz linear InGaAs PIN photodetector (PD-30, Optilab, Inc., Phoenix, AZ, USA). After detection, the signal is digitized with 12-bit resolution by a dual-channel digitizer (U5303A, Acqiris, Inc., Aulx, Switzerland). The sample light and reference light will interfere when their OPD is within the coherence length of the swept source. The depth profile of OCT image is obtained by Fourier transforming the interferometric spectrum. The system supports an axial resolution of 12.6 μm in air and a ranging depth of 1.29 mm. System sensitivity with 10.5 mW of power incident on the sample is 85 dB. The sensitivity of the system is fundamentally limited by the unstable phase noise of the custom made swept source and unbalance detection. In the proposed OCT system, the ECF instead of the commercial SMF is used as one interference arm. The elongation and compression of the ECF during directional bending process changed the optical path length. Therefore, the OPD of two arms can be adjusted with high precision using a mechanical bending device.

**Figure 4:**
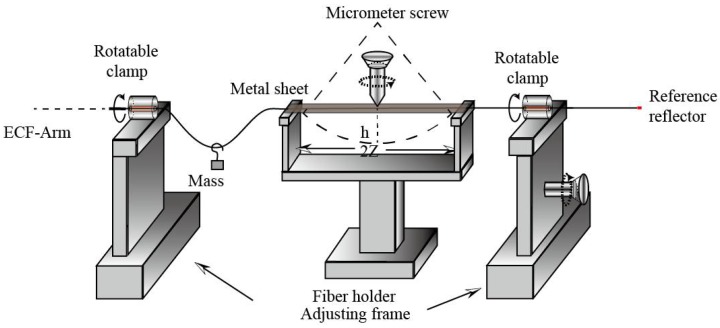
The bending setup of the ECF-arm.

The experimental setup used to carry out the bending adjustment is shown in Figure 4. The ECF-arm is placed in the middle between two fiber holders, and the distance between the two holders is 20 cm. A metal sheet is put on the two fiber holders to cover the part of ECF-arm. To keep the ECF in touch with the metal sheet during the bending process, a light mass with about 2 g is hung on the fiber between the left fiber holder and the left clamp. The bending is applied on the ECF-arm when pressing the metal sheet by a precise micrometer screw. A pair of rotatable clamps on the adjusting frames, with a division value of 5° and rotation ranges of 0–360°, are used here for changing the bending direction. The actual propagation length of the light varies with the degree of fiber bending.

## 3. Results and Discussion

**Figure 5:**
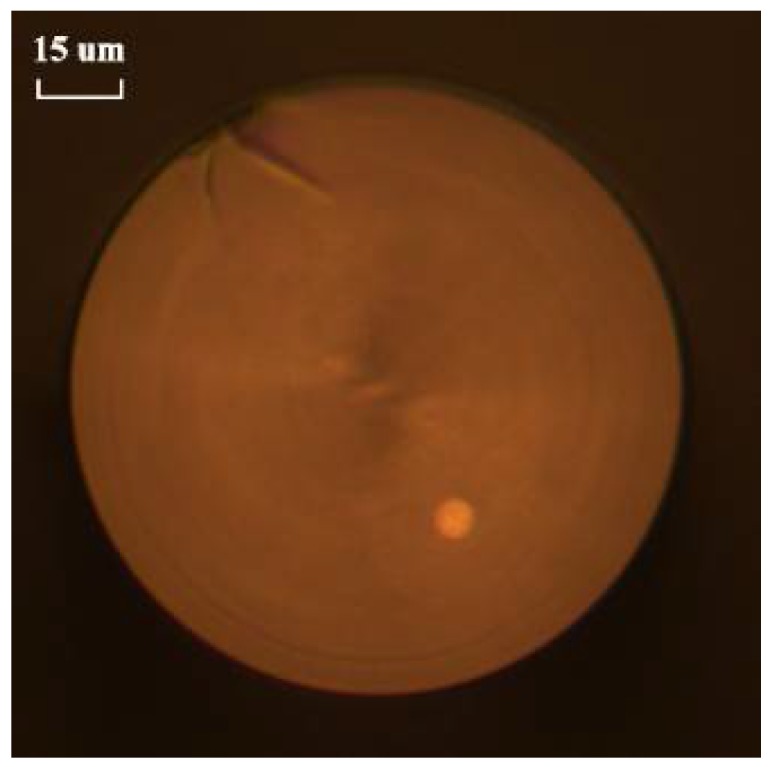
The cross-section micrograph of the ECF with a core-offset distance of ~30 μm away from the fiber’s central axis.

The cross-section micrograph of the ECF is displayed in Figure 5. It is composed of a core locating ~30 μm away from the central axis and a conventional cladding, whose diameters are about 8.5 μm and 125 μm, respectively. To understand more about the characteristics of the ECF, the birefringence was calculated and experimentally measured.

**Figure 6:**
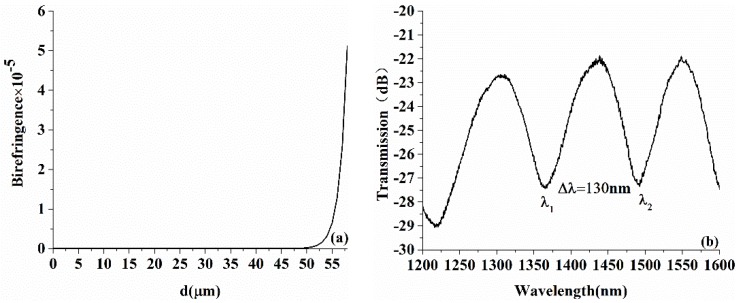

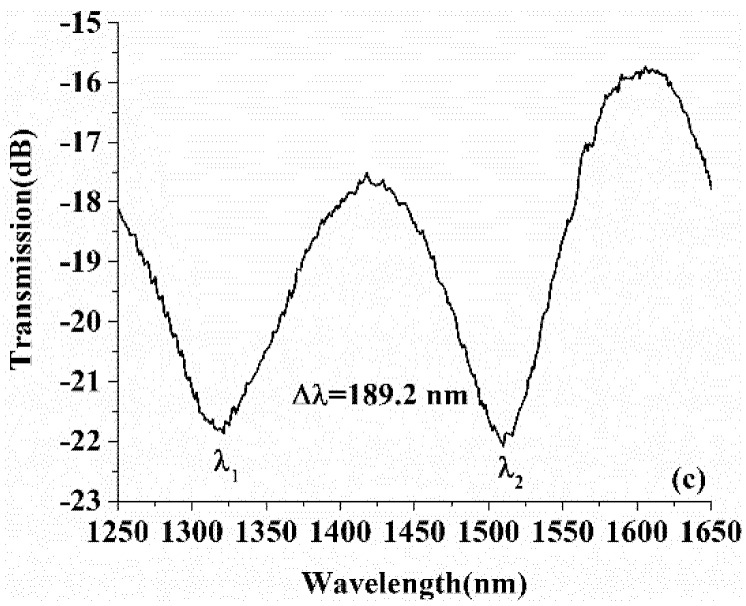
(**a**) Variation of birefringence with core eccentricity; (**b**) the experimental result for birefringence measurement of ECF; and (**c**) the experimental result for birefringence measurement of SMF.

The relationship between the birefringence of the ECF and the distance away from the central axis by numerical simulation is shown in Figure 6a. From the figure, the core-offset distance *d* makes slight effect on the birefringence of the ECF when its value is less than 50 μm. And the data shows exponential growth when it is greater than 50 μm. Figure 6b,c depict the interference signal formed by a Signac loop based on a 3 dB coupler used to measure the birefringence of ECF and SMF. Thus, the birefringence Δn can be derived as:

(4) $$\Delta n=\lambda_{1}\cdot \lambda_{2}∕\left(\Delta \lambda \cdot L\right)$$

where *L* is the length of the optical fibre being measured, which is 2.7 m in our experiment. $\lambda_{1}\;\mathrm{and}\;\lambda_{2}$ are the adjacent spectrum dips of the interference, and Δλ is the wavelength deviation. The birefringence of the ECF and SMF measured by the experiment is about 5.8 × 10^−^^6^ and 3.9 × 10^−6^ respectively, which shows that compared with those of the commercial SMF, polarization effects in the setup could be ignored.

The conventional optical fiber interfaces are mainly aimed at commercial SMF applications. Therefore, the coupling between the ECF and SMF should be taken into consideration. One of the solutions is to splice the two distinct kinds of fiber core. In our work, one end of the ECF was fusion spliced to a SMF through core alignment by a fusion splicer (FSM-80S) under manual mode. The discharge time and power level of the fusion splicer were set as 1100 ms and 24-bit, respectively. In the process, the light source and the optical power meter were used to monitor the alignment of the two cores. The SMF was connected with the light source and the ECF was connected with the optical power meter. When the power meter reading reached the maximum, the two fibers core were fully aligned. The ECF was used as one arm of the interferometer.

**Figure 7:**
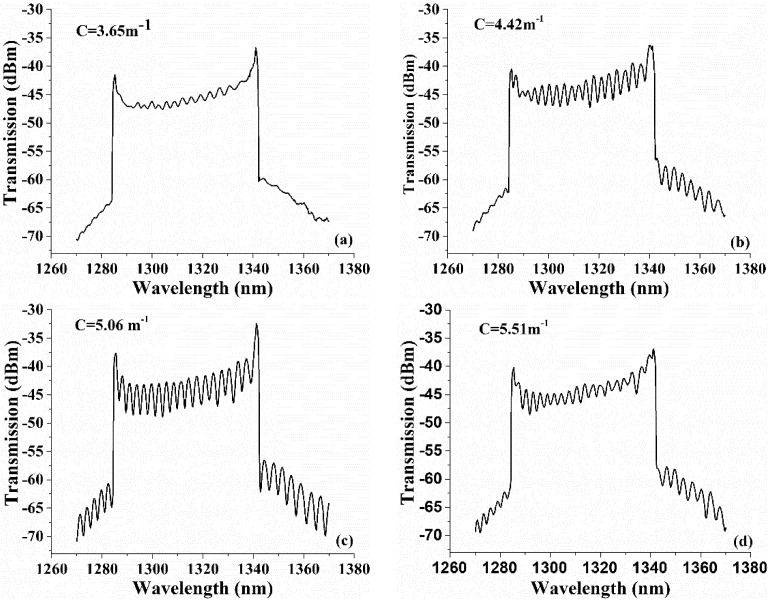
The transmitted spectrum variations against the curvature at (**a**) 3.65 m^−1^; (**b**) 4.42 m^−1^; (**c**) 5.06 m^−1^; (**d**) 5.51 m^−1^.

A mirror was used as a sample for analyzing the ECF bending responses of the system. Firstly, the ECF bending direction was adjusted by rotating the rotatable clamps to maximum sensitivity. Then, the steel sheet was pressed by rotating the precise micrometer screw so that bending was applied on the ECF. The curvature of the bending can be calculated by the formula $C=2h/\left(h^{2}+z^{2}\right)$, where h is the bending displacement of the ECF, and z is the half-distance between the two supports as shown in Figure 4. Interference fringes appeared when the curvature was increased to 3.28 m^−1^. It is obvious that the OPD $l_{r}-l_{s}$ in the sample and reference arms were matched to within the short coherence length interference but not exactly matched with good contrast. Then, different degrees of bending were applied to the ECF. Several values with distinct contrast differences were recorded. The transmitted spectrum variations against the curvature at 3.65, 4.42, 5.06 and 5.51 m^−1^ are depicted in Figure 7a–d, respectively. The length variation ΔL of the ECF were calculated as 21.90, 26.52, 30.36 and 33.06 μm, respectively. From the Figures, the fringe contrast corresponding to the four bending degrees were tested as 1.23, 4.12, 6.27 and 2.75 dB, respectively. The interference fringe contrasts alter from 1.23 to 6.27 dB with the variations of curvature. This is because the elongation of the ECF during the bending process changed the optical path length in ECF to be better matched. Figure 7d shows an obviously decrease in contrast. It indicates that that depth is close to coherence length. Therefore, the length of the reference arm can be adjusted by adjusting the micrometer. As a result, the OPD of two arms is adjusted with high precision just using a mechanical bending equipment and no power supply required, which simplifies the structure of the OCT system, improves the fringe contrast and performance of image reconstruction for different depth sample. The obtained length variations in the experiment are of the order of tens of micrometers. It makes sense for light source with a low coherence length. Besides, we can increase the bending length of ECF or distance between the eccentric core and the neutral plane to increase the attainable range for large imaging depth range OCT systems.

**Figure 8:**
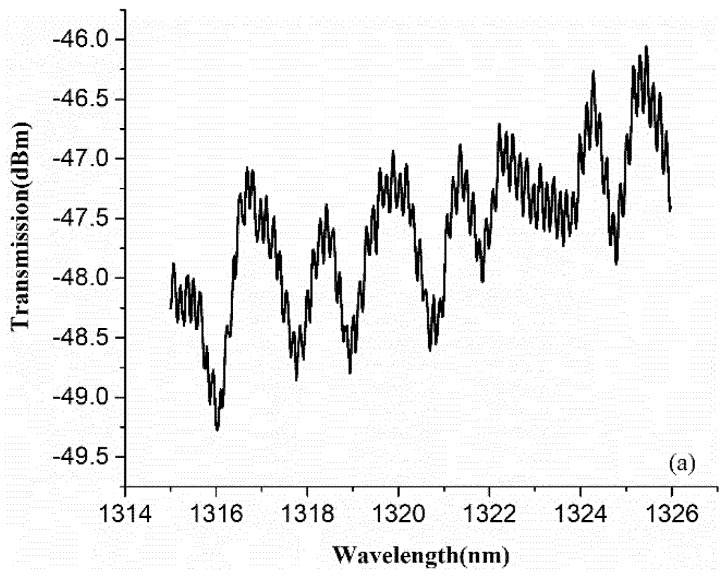

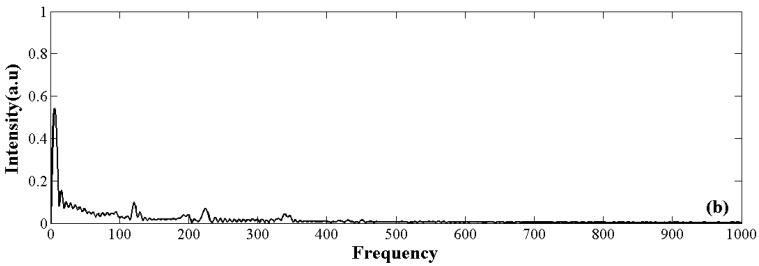
(**a**) Transmitted spectrum of four pieces of overlapping glass slides measured by the proposed OCT system (**b**) FFT spectrum of four pieces of overlapping glass slides in the proposed OCT system.

Four pieces of overlapping glass slides were then used as a sample for tomography. Contrast-enhanced fringe was obtained by adjusting the degree of ECF bending. Figure 8a,b show the transmitted spectrum and the corresponding FFT spectrum of the sample in the proposed OCT system, respectively. As shown in Figure 8a, the envelope is the interference signal between sample and reference reflector, and the high frequency signal in the envelope is the autocorrelation interference among each reflective surface. The frequencies for each reflective surface can be clearly observed in Figure 8b.

**Figure 9:**
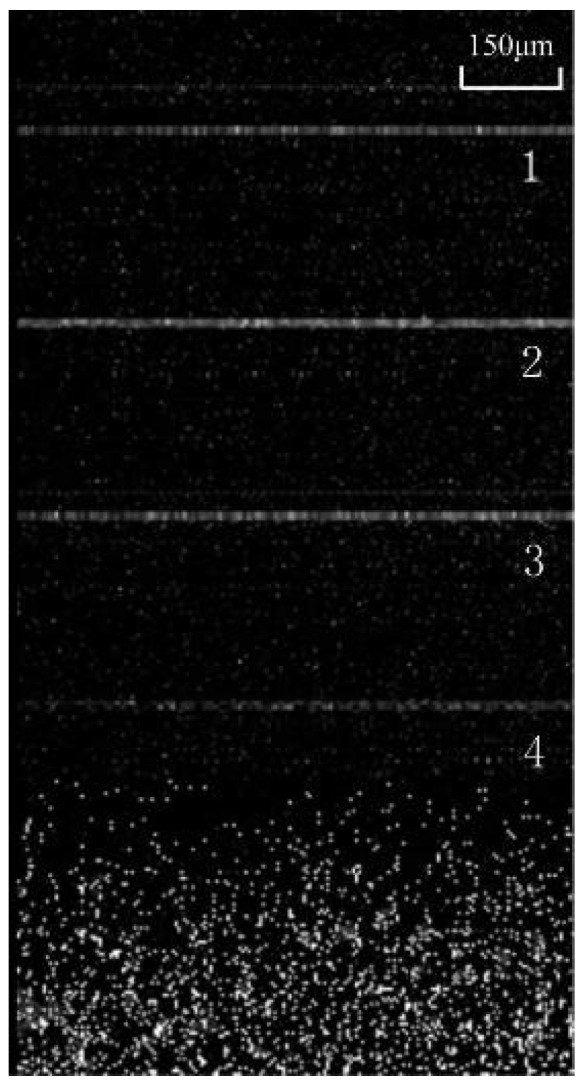
The 2D tomography of the four pieces of overlapping glass slides in the proposed OCT system.

The effect of the proposed OCT system is illustrated here. It can adjust the OPD of two arms and has improvement in the fringe contrast. Figure 9 shows the cross-sectional OCT image of the four pieces of overlapping glass slides in the proposed OCT system obtained at 2000 (2 kHz) axial scans per second. Four pieces of glass slides can be observed.

## 4. Conclusions

In conclusion, a MZI using and ECF design for OCT is proposed and demonstrated. The ECF is used as one arm of the MZI. Interference signal changes with the variations of curvature. Interference fringes appear when the curvature is increased to 3.28 m^−1^. The interference fringe contrast alters from 1.23 to 6.27 dB with the increase variation of curvature. This is because the elongation of the ECF during the bending process change the optical path in ECF. Therefore, the proposed implement with a simple structure can replace the reference delay line to form an all-fiber configuration. Besides, four pieces of overlapping glass slides with four reflective surfaces as sample is experimentally measured as well, which demonstrates the feasibility of the system. The 2D tomography of the four pieces of overlapping glass slides in the proposed OCT system is also obtained. The proposed system is compact, economical, and adjustable with higher precision than traditional system.
